# PPARγ Mediates the Anti-Epithelial-Mesenchymal Transition Effects of FGF1^ΔHBS^ in Chronic Kidney Diseases via Inhibition of TGF-β1/SMAD3 Signaling

**DOI:** 10.3389/fphar.2021.690535

**Published:** 2021-06-03

**Authors:** Dezhong Wang, Tianyang Zhao, Yushuo Zhao, Yuan Yin, Yuli Huang, Zizhao Cheng, Beibei Wang, Sidan Liu, Minling Pan, Difei Sun, Zengshou Wang, Guanghui Zhu

**Affiliations:** ^1^ Institute of Life Sciences and Engineering Laboratory of Zhejiang Province for Pharmaceutical Development of Growth Factors, Wenzhou University, Wenzhou, China; ^2^ The First Affiliated Hospital of Wenzhou Medical University, Wenzhou, China; ^3^ School of Pharmaceutical Sciences, Wenzhou Medical University, Wenzhou, China; ^4^ The Second Affiliated Hospital and Yuying Children’s Hospital of Wenzhou Medical University, Wenzhou, China

**Keywords:** fibrosis, FGF1, PPARγ, chronic kidney disease, epithelial-mesenchymal transition

## Abstract

Podocytes are essential components of the glomerular basement membrane. Epithelial-mesenchymal-transition (EMT) in podocytes results in proteinuria. Fibroblast growth factor 1 (FGF1) protects renal function against diabetic nephropathy (DN). In the present study, we showed that treatment with an FGF1 variant with decreased mitogenic potency (FGF1^ΔHBS^) inhibited podocyte EMT, depletion, renal fibrosis, and preserved renal function in two nephropathy models. Mechanistic studies revealed that the inhibitory effects of FGF1^ΔHBS^ podocyte EMT were mediated by decreased expression of transforming growth factor β1 via upregulation of PPARγ. FGF1^ΔHBS^ enhanced the interaction between PPARγ and SMAD3 and suppressed SMAD3 nuclei translocation. We found that the anti-EMT activities of FGF1^ΔHBS^ were independent of glucose-lowering effects. These findings expand the potential uses of FGF1^ΔHBS^ in the treatment of diseases associated with EMT.

## Introduction

Podocytes are an essential part of the glomerular filtration barrier. Their injury leads to several glomerular diseases that develop to end-stage renal disease (ESRD) (Fishel Bartal et al., 2020). Podocytes are highly specialized epithelial cells; epithelial-mesenchymal-transition (EMT) in podocytes has been observed in chronic kidney disease (CKD) (Liu, 2010; Asfahani et al., 2018; Yin et al., 2018). The expression of nephrin, podocin, and ZO-1 was decreased during podocyte EMT, resulting in the abnormal glomerular basement membrane (GBM) and fibrosis (He et al., 2011; Choi et al., 2020). Owing to its pivotal role in renal function, podocyte homeostatic regulation is a promising strategy for treating CKD.

Studies confirmed that EMT is an essential mechanism of the accumulation and deposition of the extracellular matrix that leads to renal fibrosis (Wynn and Ramalingam, 2012; Kang et al., 2020). Transforming growth factor-β1 (TGF-β1) is the most potent EMT inducer, and enhanced expression of TGF-β1 was noted in renal tissues in the context of CKD (Chen et al., 2021). Biological functions induced by TGF-β1 depend on accelerating the phosphorylation of Smad3 and nuclei translocation that activates the transcription of target genes (Meng et al., 2016). Bone morphogenetic protein 2 (BMP2) is a sub-member of the TGF-β superfamily. Defective signaling transduction in this pathway is present in hereditary, idiopathic, and other forms of CKD (Orriols et al., 2017). BMP2 antagonizes the TGF-β1/TGF-βR pathway through peroxisome proliferator-activated receptor γ (PPARγ), which participates in cardiovascular homeostasis and glucose metabolism (Tyagi et al., 2011; Chen et al., 2012; Calvier et al., 2017). Several lines of evidence suggested podocyte protection *via* activation of PPARγ (Kanjanabuch et al., 2007; Henique et al., 2016; Zhou et al., 2017). Nevertheless, the side effects of PPARγ agonists such as thiazolidinedione (TZD) limit its use in CKD treatment.

Fibroblast growth factor 1 (FGF1) mediates wound healing, angiogenesis, embryonic development, and neurogenesis (Xie et al., 2020). Recently, FGF1 was found to function as a critical metabolic hormone that is pivotal for regulating insulin sensitivity, glycemic control, and nutrient stress (Beenken and Mohammadi, 2009; Gasser et al., 2017). FGF1 treatment increased insulin sensitization, maintained normoglycemia, and prevented diabetic complications, including hepatic steatosis and podocyte injury (Suh et al., 2014; Gasser et al., 2017; Liang et al., 2018; Lin et al., 2020). Nevertheless, the underlying mechanism of FGF1 or its variant’s protective effects on podocyte EMT remains unclear.

FGF1 exerts its biological function *via* heparin sulfate-assisted FGF receptor dimerization and downstream signal transduction. Previously, we obtained an FGF1 variant (FGF1^ΔHBS^) by replacing 3 residues from heparin sulfate binding site (Lys127Asp, Lys128Gln and Lys133Val) that exhibited full metabolic capacity and much less proliferative potential than wild-type FGF1 (Huang et al., 2017). We employed two murine models of CKD to investigate the protective role and underlying mechanisms preventing podocyte EMT.

## Materials and Methods

### Regents and Antibodies

Doxorubicin (adriamycin) was purchased from Selleck (Cat# S1208). RPMI-1640 medium and penicillin-streptomycin were purchased from Gibco. Fetal bovine serum (FBS) was purchased from ScienCell. Hydroxyproline content assay kit was purchased from Solarbio (Cat# BC0255). Mouse interferon was purchased from Cell Signaling Technology (Cat# 39127). PPARγ siRNA was purchased from Santa Cruz (Cat# sc-29456). Serum levels of blood urea nitrogen (BUN), ALB and creatinine were measured using assay kits according to the manufacturer’s instructions (Jiancheng, Nanjing, China). Kits for Sirius red staining, Masson trichrome staining and hematoxylin and eosin (H&E) were purchased from Beyotime Biotech (Nantong, China). BCA kits were used to measure protein concentration (Transgen, Cat# DQ111). The SuperSignal™ West Pico PLUS (Thermo, Cat# 34577) was chosen to visualize the immunoreactive bands.

The following antibodies were used to measure the proteins of interest: COL 1 (Abcam; Cat# ab34710, dilution: 1:800), COL 4 (Proteintech; Cat# 55131-1-AP, dilution: 1:800), α-Smooth Muscle Actin (Abcam; Cat# 19245, dilution: 1:1,000), TGF-β1 (Abcam; Cat# ab215715; dilution: 1:800), phospho-SMAD3 (Cell Signaling; Cat# 9520, dilution: 1:1,000. Abcam, Cat# ab52903, dilution: 1:100), SMAD3 (Proteintech; Cat# 66516-1, dilution: 1:1,000), GAPDH (Cell Signaling; Cat# 5174, dilution: 1:1,000), PPARγ (Santa Cruz; Cat# sc-7273, dilution: 1:1,000), goat anti-rabbit secondary antibody (Abcam; Cat# ab150080, dilution: 1:200), goat anti-mouse secondary antibody (Abcam; Cat# ab6717, dilution: 1:200), HRP-conjugated antibodies (Cell Signaling; Cat# 7074 or 7076, 1:3,000), and biotinylated antibody (Zhongshan Golden Bridge; Cat# ZB-2010, 1:80). Transfection reagent was purchased from Invitrogen (Cat# 13778030). FGF1^ΔHBS^ was expressed and purified as described (Wang et al., 2019).

### Cell Culture

Cell culture and treatment were performed as described (Wang et al., 2019). Briefly, conditionally immortalized mouse podocyte cell line were cultured at 33°C for proliferation. Cell differentiation was induced for 10 days at 37°C, and then starved for 12 h and pretreated with FGF1^ΔHBS^ (100 ng/ml) for 1 h. Then the cells were incubated in high glucose (HG, 25 mM) (with D-mannitol as an osmotic control) or ADR (0.5 μ g/ml) for 12 h. For PPARγ knockdown experiments, specific siRNA was transfected using transfection reagent Lipofectamine 3000 according to the manufacturer’s protocol.

### Animals

8 week-old male d*b/*d*b* mice, their d*b/m* littermates, and male BALB/c mice were purchased from the GemPharmatech Co., Ltd., (Nanjing China). Animals were maintained in a controlled environment (12 h light/dark cycle at 23°C) with free access to food and water. The experiments were performed following the National Institutes of Health guidelines and with approval from the Animal Care and Use Committee of Wenzhou Medical University, China.

For the DN model, d*b/*d*b* mice were intraperitoneally (i.p.) injected with FGF1^ΔHBS^ at 0.5 mg/kg body weight every other day for 8 weeks while d*b/m* and d*b/*d*b* mice were received 0.9% normal saline as controls. Blood glucose levels were measured using a blood glucose monitor (Roche).

For the adriamycin-induced nephropathy (AN) model, mice were injected with a single dose of ADR (11 mg/kg) through the tail vein. FGF1^ΔHBS^ (0.5 mg/kg body weight) or normal saline was administered i.p. every other day starting one week before ADR injection and lasting for 5 weeks. Metabolic cages (TSE Systems, MO) were chosen to collect mice urine for 24 h.

### Histological Analysis

Renal tissues were fixed and sectioned at 5–6 μm thickness. For immunohistochemistry analysis, sections were incubated with antibody overnight and incubated with the biotinylated antibody for 1 h and stained with DAPI. Stained sections were evaluated for histopathological damage (Nikon, Japan).

For transmission electron microscope analysis, renal samples were fixed using a triple aldehyde fixative overnight at 4°C. Specimens were incubated with uranyl acetate and embedded in epoxy resin after rinsing. Sections were stained and observed under an electron microscope (JEOL, Japan).

For immunofluorescence staining, renal tissues or cells were fixed with 4% paraformaldehyde for15 min, permeabilized with 0.1% Triton X-100 for 10 min, and incubated with anti-PPARγ and anti-SMAD3 antibody overnight at 4°C in a humidified atmosphere in the dark. Following incubation with a secondary antibody, the cells were evaluated using a Nikon confocal microscope (Nikon, Japan).

### Real-Time PCR Analysis

MiniBEST Universal RNA Extraction Kit (Takara, Cat# 9767) were used to extract total RNA and RNA was reverse transcribed using PrimeScript™ RT Master Mix (Takara, Cat# RR036A). Real-time PCR was conducted using a QuantStudio3 system with TB Green qPCR Master Mix (Clontech, Cat# 639676). Primers are listed in Supplementary Table S1.

### Western Blot Analysis

Renal tissues (25–40 mg) or cells were lysed and protein concentrations were determined using the BCA kit (Thermo, Cat# 23225) per the manufacturer’s introduction. Equal amounts of samples were subjected to electrophoresis, transferred to nitrocellulose membranes, and blocked. After incubation with antibodies, the blots were incubated using commercial kits to visualize. Densitometric analysis was performed using ImageJ (NIH, United States of America).

### Statistical Analysis

All data were expressed as mean ± SEM. *In vitro* experiments were repeated in triplicate (biological repeat) for each experiment. One-way ANOVA followed by the Tukey post hoc test was used to compare more than two groups’ mean values. Two-way ANOVA followed by Turkey post hoc test was used to compare the effects of PPARγ knockdown in response to FGF1^∆HBS^ treatment. GraphPad Prism was used to analysis the statistical tests. *p*-values less than 0.05 were considered statistically significant.

## Results

### FGF1^ΔHBS^ Prevents Renal Remodeling in *db/db* Mice

**FIGURE 1:**
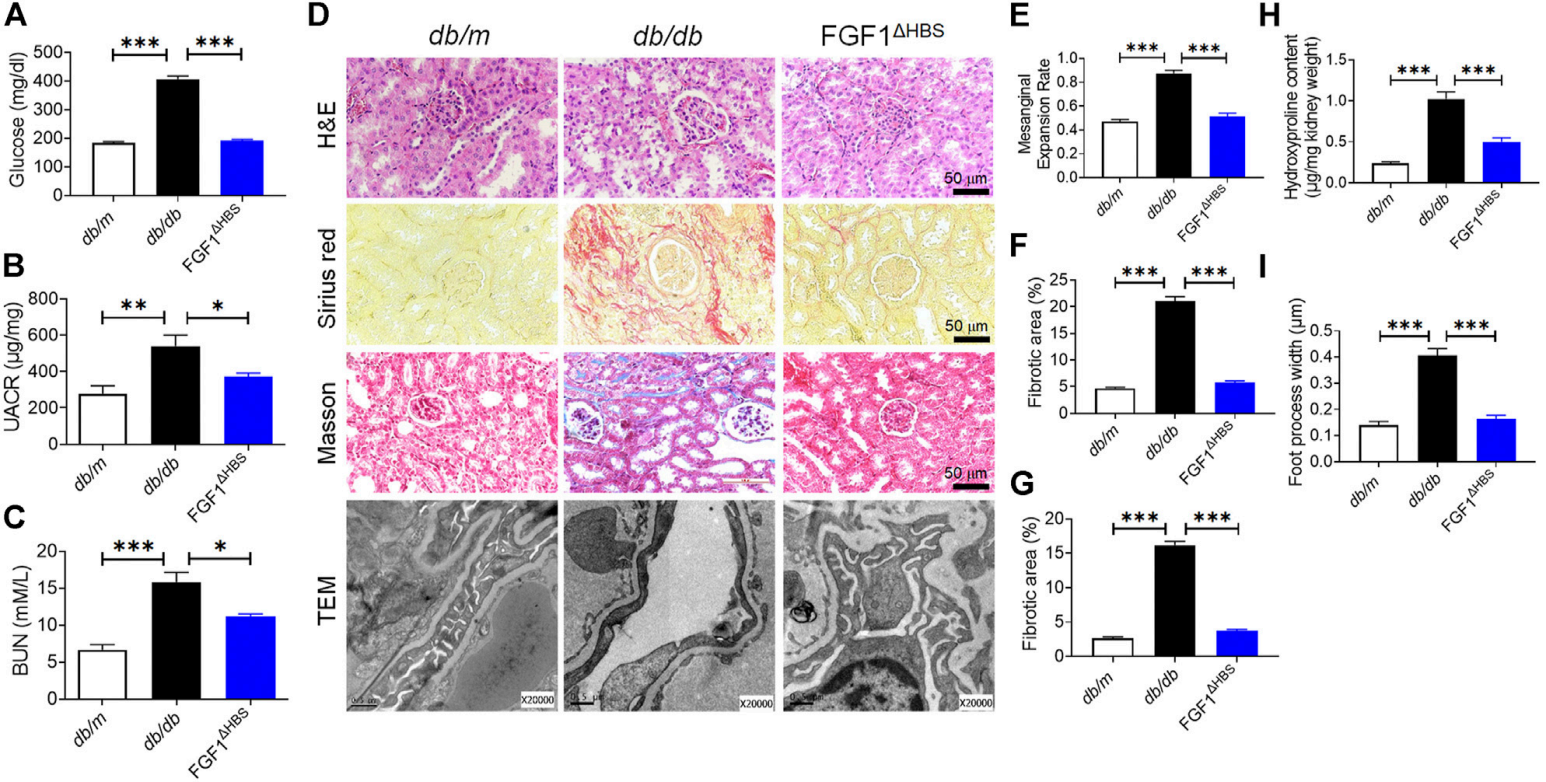
FGF1^ΔHBS^ prevented renal remodeling and dysfunction in d*b/*d*b* mice. **(A)** Blood glucose levels. **(B)** Urine albumin-to-creatinine ratio (UACR). **(C)** Blood urea nitrogen (BUN) levels. **(D)** Representative images of hematoxylin and eosin (H&E) staining, Sirius red staining, Masson’s trichrome staining and transmission electron microscopy (TEM) images of renal tissues. **(E)** Quantification of mesangial expansion. **(F)** Quantification of the fibrotic area in Sirius red staining. **(G)** Quantification of the fibrotic area in Masson staining. **(H)** Hydroxyproline content in renal tissues. **(I)** Quantification of podocyte foot process effacement. Data are presented as the mean ± SEM (*n* = 6); *p < 0.05, **p < 0.01, ***p < 0.001.

To explore the anti-fibrotic effects of FGF1^ΔHBS^ against diabetes-induced CKD, d*b/*d*b* mice received FGF1^ΔHBS^ every other day for 8 weeks. As shown in Figure 1A, FGF1^ΔHBS^ decreased blood glucose in d*b/*d*b* mice, consistent with our previous findings (Wang et al., 2021). The increase of urine albumin-to-creatinine ratio (UACR) was ameliorated in FGF1^ΔHBS^-treated group and serum levels of BUN were lower following FGF1^ΔHBS^ treatment as well (Figures 1B,C).

DN is characterized by mesangial expansion, collagen accumulation, and podocyte loss (Alicic et al., 2021). H&E staining revealed that mesangial expansion was relieved by FGF1^ΔHBS^ treatment (Figures 1D,E), and renal fibrosis was significantly reduced (Figures 1D–H). Podocyte injury is associated with proteinuria, and podocyte loss is the primary starting point of glomerular damage (Koga et al., 2015). Disruption of podocyte foot processes and thickening of basement membranes were found in d*b/*d*b* mice (Figures 1D–I). These pathological findings were ameliorated in the FGF1^ΔHBS^-treated group (Figures 1D–I). These data suggest that FGF1^ΔHBS^ mitigates renal remodeling, fibrosis, and podocyte injury in diabetic mice.

### FGF1^ΔHBS^ Decreases Expression of TGF-β1 and SMAD3 Phosphorylation in Renal Tissues of Diabetic Mice

**FIGURE 2:**
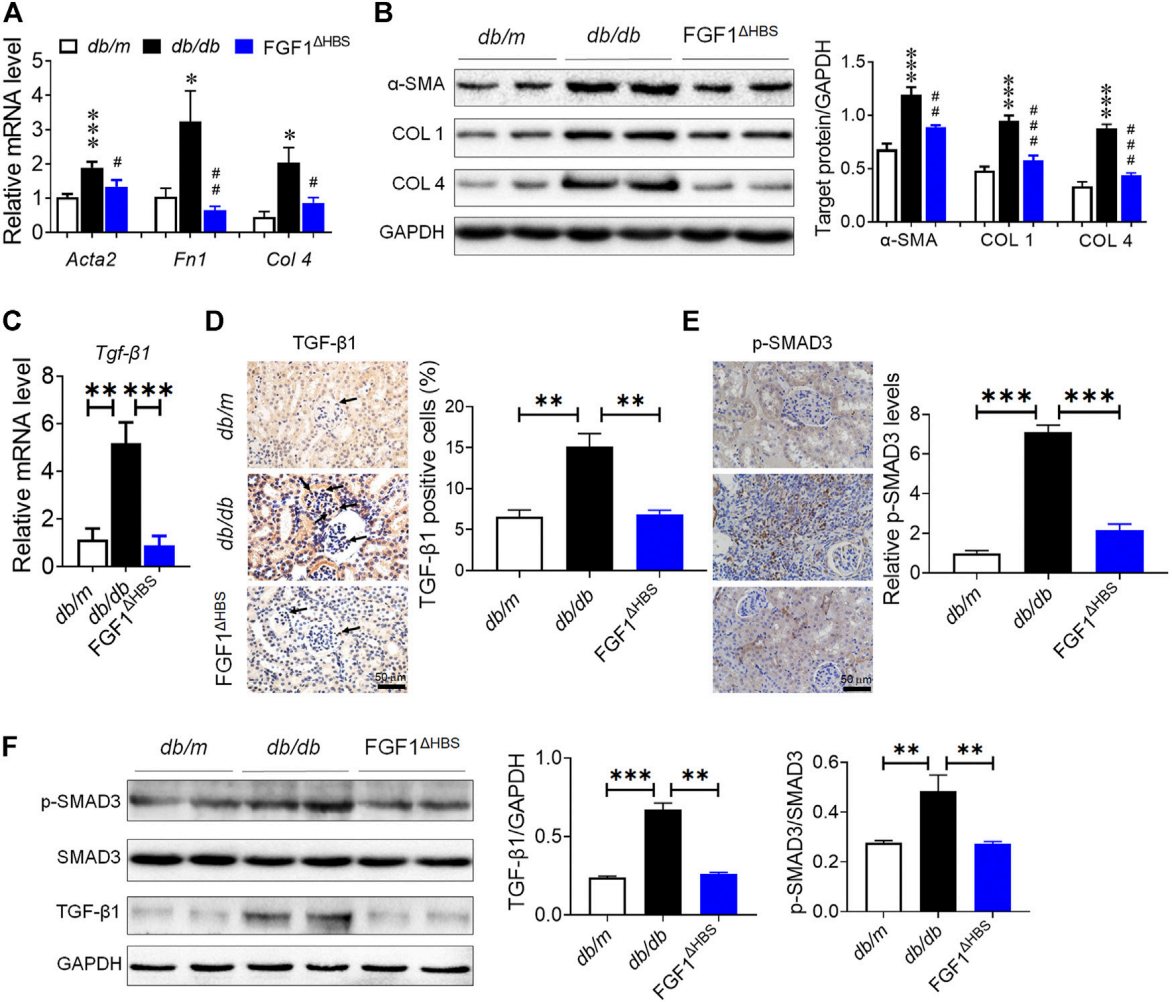
FGF1^ΔHBS^ suppressed renal fibrosis and TGF-β1 signaling in d*b/*d*b* mice. **(A)** Real-time PCR analysis of *Acta2*, *Fn1*, and *Col 4* mRNA expression. **(B)** Expression levels of α-SMA, COL 1, and COL 4 as determined by western blot analysis and quantitation using ImageJ. **(C)** Real-time PCR analysis of TGF-β1 mRNA expression levels. **(D)** Representative images of TGF-β1 immunohistochemical staining of renal tissues and quantitation using ImageJ. (**E**) Representative images of phosphorylated SMAD3 immunohistochemical staining of renal tissues and quantitation using ImageJ. **(F)** Expression levels of phosphorylated SMAD3, SMAD3, and TGF-β1 as determined by western blot analysis and quantitation using ImageJ. Data are presented as the mean ± SEM (*n* = 6); Panels A and B, *p < 0.05, ***p < 0.001 vs. d*b/m*; ^#^ *p* < 0.05, ^##^ *p* < 0.01, ^###^ *p* < 0.001 vs. d*b*/d*b*; Panels C–F, **p < 0.01, ***p < 0.001.

Given the significantly decreased deposition of extracellular matrix in kidneys by FGF1^ΔHBS^ treatment, we measured mRNA expression levels of fibrotic genes. As shown in Figure 2A, there was diabetes-induced upregulation of *Acta2* (an indicator of fibrosis), *Fn1* (participates in extracellular matrix formation), and *Col 4* (main component of the glomerular basement membrane) in renal tissues. FGF1^ΔHBS^ inhibited the mRNA levels of these genes (Figure 2A). These results were further confirmed by western blot analysis in which protein levels of α-SMA, COL 1, and COL 4 were increased in renal tissues from d*b/*d*b* mice and were remarkably restored by FGF1^ΔHBS^ treatment (Figure 2B).

Since TGF-β1 participates in promoting the deposition of extracellular matrix, podocyte EMT, and apoptosis (Liu, 2004), we used immunohistochemistry to analyze the expression of TGF-β1. We first measured the mRNA expression of TGF-β1. As shown in Figure 2C, FGF1^ΔHBS^ decreased diabetes-induced upregulation of Tgf-β1 in renal tissues. Increased expression of TGF-β1 in the glomeruli was observed in buffer-treated mice, and FGF1^ΔHBS^ treatment substantially reduced positive cell numbers (Figure 2D). SMAD3 mediated the intracellular signaling of TGF-β1 by shuttling into the nuclei and promoting transcription of target genes for which phosphorylation is essential (Le et al., 2020). We next analyzed the phosphorylation levels of SMAD3 by immunohistochemistry staining (Figure 2E) and found that the increased phosphorylation of SMAD3 was strongly suppressed, along with decrease of protein expression of TGF-β1 by FGF1^ΔHBS^ treatment (Figures 2E,F). These data suggest that FGF1^ΔHBS^ prevents renal fibrosis and podocytes injury *via* downregulation of TGF-β1 and SMAD3 phosphorylation expression.

### PPARγ Mediated the Anti-EMT Effects of FGF1^ΔHBS^ in Diabetes

**FIGURE 3:**
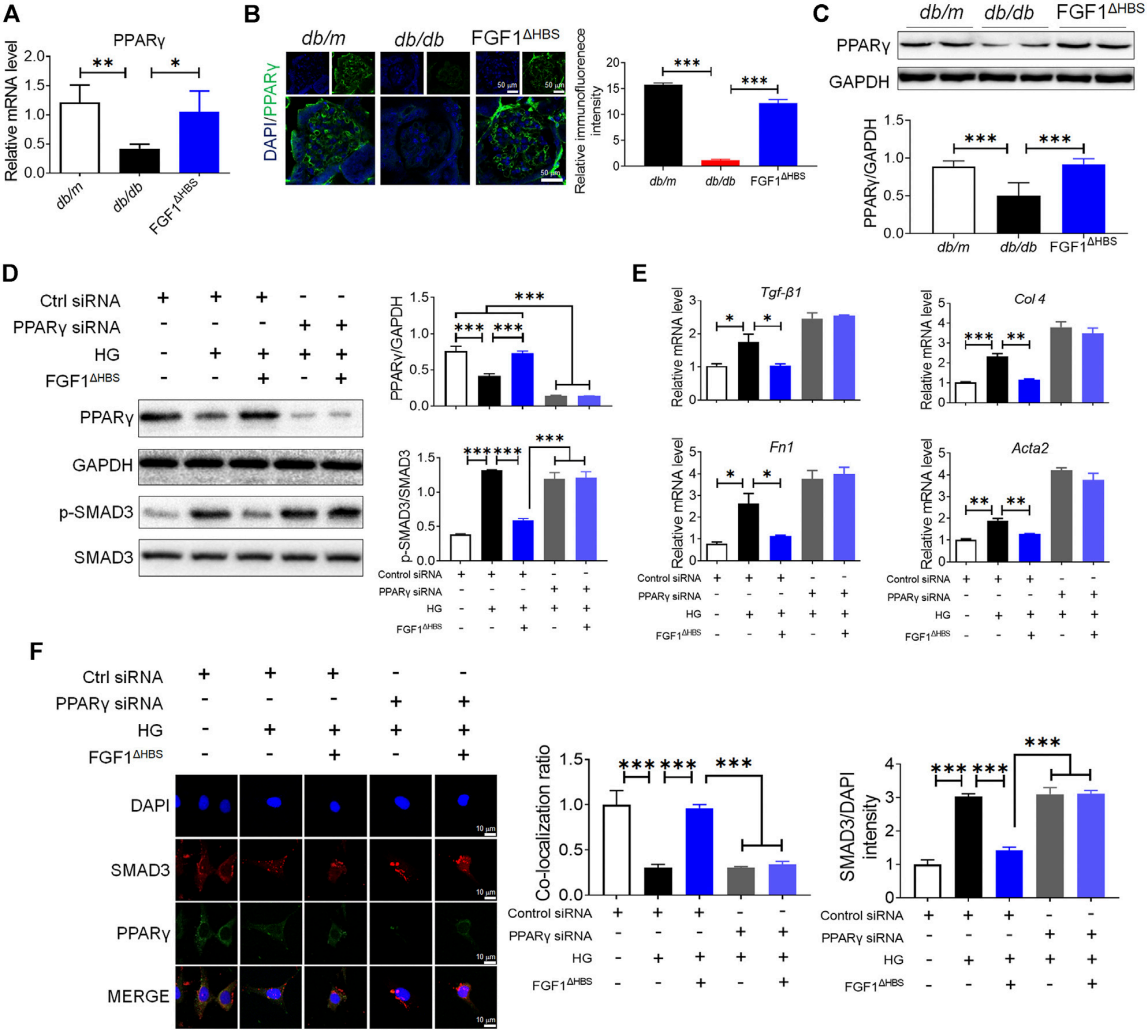
PPARγ mediated the protective effects of FGF1^ΔHBS^ on podocyte EMT in diabetic conditions. **(A)** Real-time PCR analysis of PPARγ mRNA expression. **(B)** Representative images and quantitation of immunofluorescence staining for PPARγ. **(C)** Expression levels of PPARγ as determined by western blot analysis and quantitation using ImageJ. **(D)** Phosphorylation levels of SMAD3 and protein expression of PPARγ as determined by western blot analysis and quantitation using ImageJ. **(E)** Real-time PCR analysis of *TGF-β1*, *Fn1*, *Col 4*, and *Acta2* mRNA expression. (**F**) Representative images and quantitation of immunofluorescence staining of SMAD3 and PPARγ. In panels **(A–C)**, data are presented as the mean ± SEM (*n* = 6). In panels **(D–F)**, data from three independent measurements are presented as the mean ± SEM; *p < 0.05, **p < 0.01, ***p < 0.001.

The crosstalk between TGF-β and BMP signaling pathways tunes the accumulation of extracellular matrix and EMT (Munoz-Felix et al., 2015; Kim et al., 2020). Several lines of evidence suggest that PPARγ participates in maintaining podocyte homeostasis and renal function (Agrawal et al., 2021). We then measured mRNA levels of PPARγ in d*b/*d*b* mice. As shown in Figure 3A, diabetes downregulated renal PPARγ expression in d*b/*d*b* mice and FGF1^ΔHBS^ treatment significantly increased PPARγ transcription. Consistent with these findings, immunofluorescence confirmed reduced expression of PPARγ in glomeruli of d*b/*d*b* mice (Figure 3B). FGF1^ΔHBS^ enhanced fluorescence intensity (Figure 3B). The protein expression of PPARγ was also measured using western blot, confirming upregulation by FGF1^ΔHBS^ treatment (Figure 3C).

Podocyte depletion caused by EMT is one of the critical determinants of CKD (Dai et al., 2017). To determine the role of PPARγ in FGF1^ΔHBS^-preserved podocytes, we used specific PPARγ siRNA to knock down protein expression. We found that HG treatment increased the phosphorylation of SMAD3 and downregulated PPARγ expression (Figure 3D). Podocytes treated with FGF1^ΔHBS^ inhibited SMAD3 phosphorylation in a PPARγ-dependent manner (Figure 3D). Real-time PCR showed that the expression of *Tgf-β1*, *Col 4*, *Fn1*, and *Acta2* were attenuated by FGF1^ΔHBS^ treatment under HG challenge, while these inhibitory effects were abolished in the presence of PPARγ siRNA (Figure 3E). The inhibitory effects of FGF1^ΔHBS^ on nuclei translocation of SMAD3 was PPARγ dependent (Figure 3F). Enhanced interaction between SMAD3 and PPARγ was also observed after FGF1^ΔHBS^ treatment (Figure 3F). Taken together, these data suggest that FGF1^ΔHBS^ protects podocytes from HG-induced EMT and injury, highlighting the importance of PPARγ in the maintenance of podocyte homeostasis and renal function.

### FGF1^ΔHBS^ Inhibited Renal Remodeling in Adriamycin-Induced CKD

**FIGURE 4:**
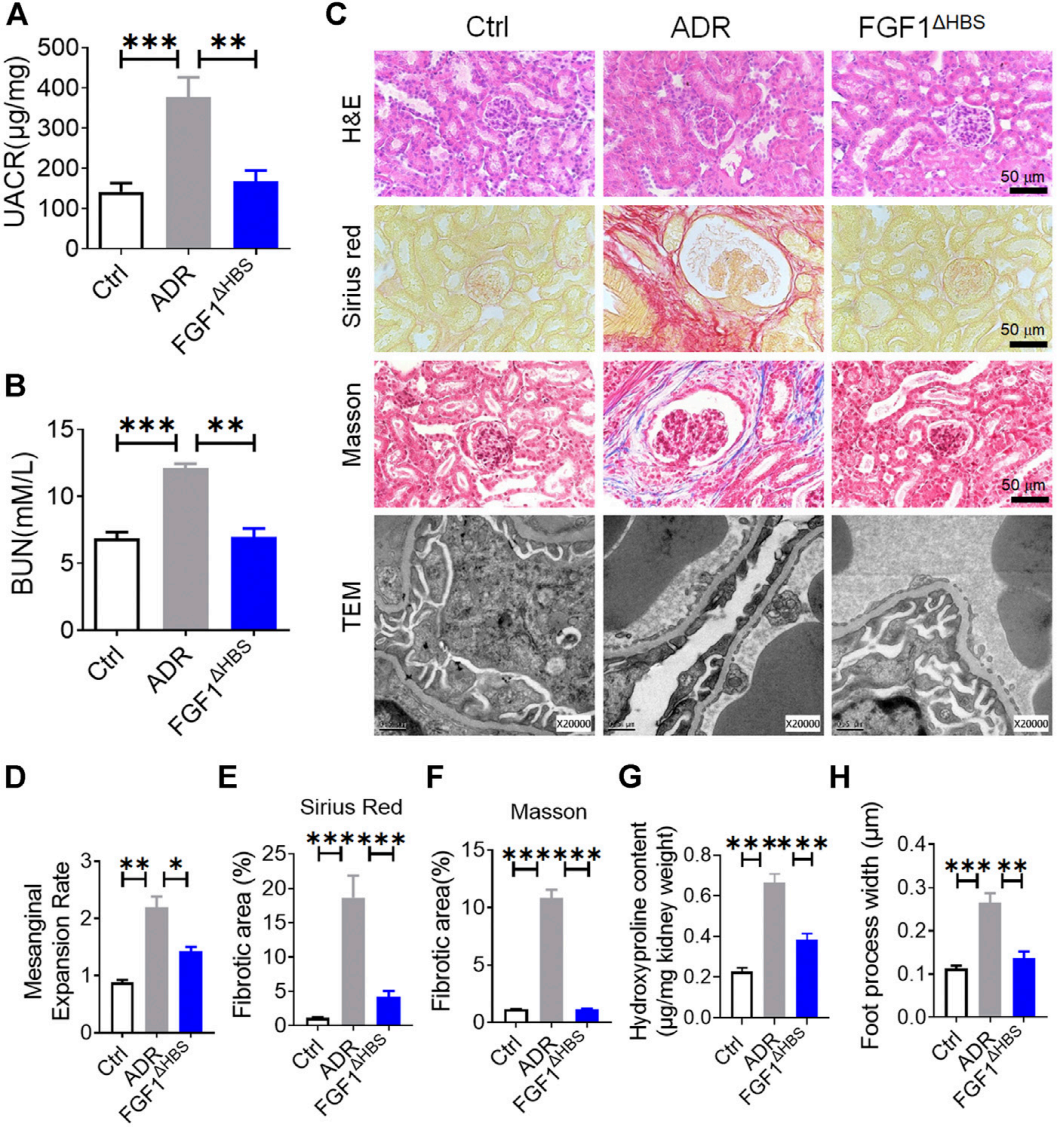
FGF1^ΔHBS^ inhibited ADR-induced renal remodeling and dysfunction. **(A)** Urine albumin-to-creatinine ratio (UACR). **(B)** Blood urea nitrogen (BUN) levels. **(C)** Representative images of hematoxylin and eosin (H&E) staining, Sirius red staining, Masson’s trichrome staining, and transmission electron microscopy (TEM) images of renal tissues. **(D)** Quantification of mesangial expansion. **(E)** Quantification of the fibrotic area in Sirius red staining. **(F)** Quantification of the fibrotic area in Masson staining. **(G)** Hydroxyproline content in renal tissues. **(H)** Quantification of podocyte foot process effacement. Data are presented as the mean ± SEM (*n* = 6); *p < 0.05, **p < 0.01, ***p < 0.001.

To explore whether the inhibitory effect of podocyte EMT by FGF1^ΔHBS^ applied to other types of CKD, we used an ADR-induced nephropathy model to investigate the anti-EMT effects of FGF1^ΔHBS^. Consistent with our previous findings (Wang et al., 2019), renal function was restored by FGF1^ΔHBS^ treatment, as evidenced by decreased UACR and BUN levels (Figures 4A,B). ADR-induced mesangial expansion was inhibited in the FGF1^ΔHBS^-treated group (Figures 4C,D). In addition, tissue remodeling was significantly prevented as renal fibrosis and collagen deposition was attenuated, and foot process loss was alleviated (Figures 4C,E–H).

### FGF1^ΔHBS^ Inhibited ADR-Induced the Upregulation of TGF-β1 and Phosphorylation of SMAD3

**FIGURE 5:**
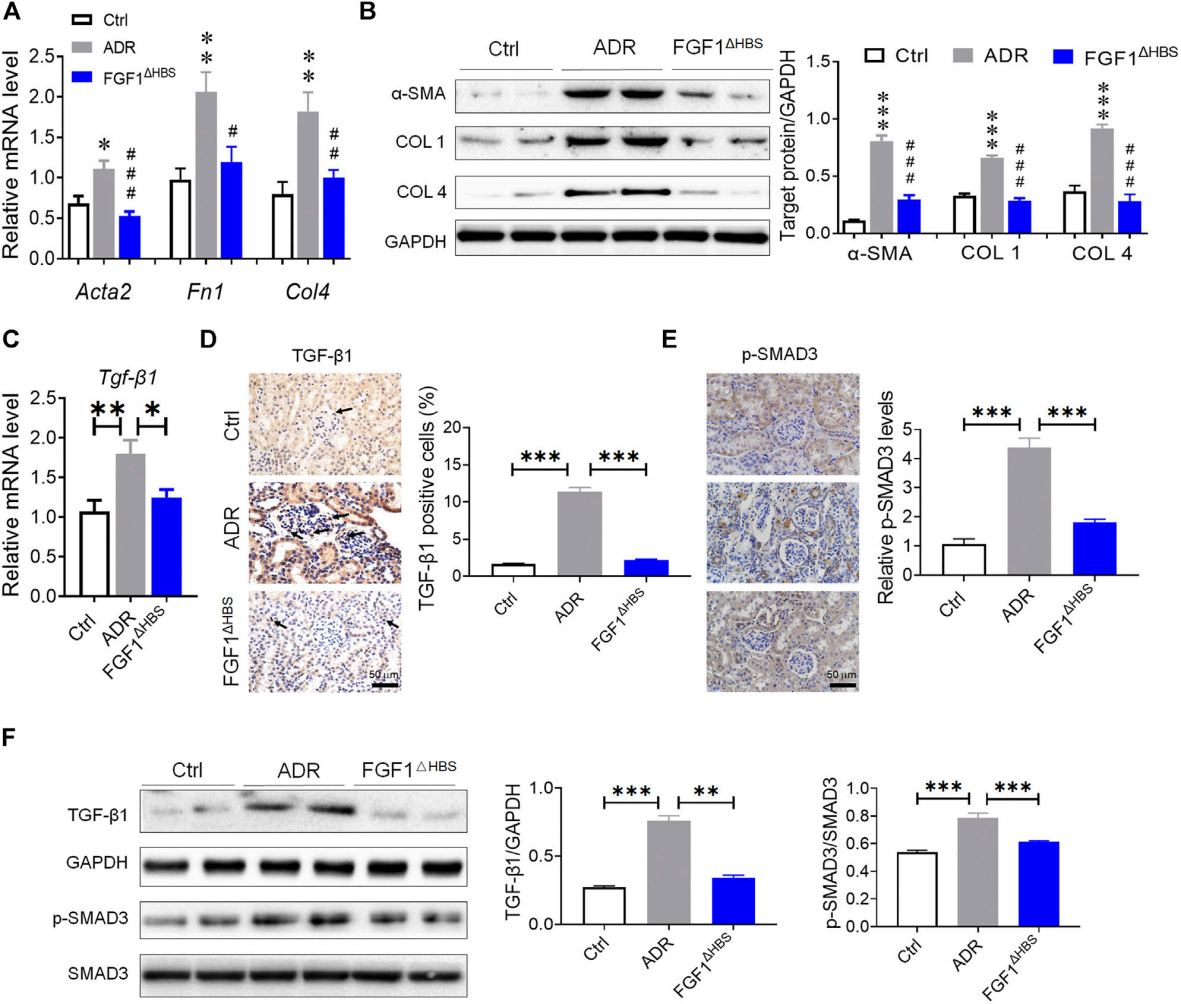
FGF1^ΔHBS^ inhibited ADR-induced the upregulation of TGF-β1 and phosphorylation of SMAD3. **(A)** Real-time PCR analysis of *Acta2*, *Fn1*, and *Col 4* mRNA expression. **(B)** Expression levels of α-SMA, COL 1, and COL 4 as determined by western blot analysis and quantitation using ImageJ. **(C)** Real-time PCR analysis of TGF-β1 mRNA expression levels. **(D)** Representative images of TGF-β1 immunohistochemical staining of renal tissues and quantitation using ImageJ. **(E)** Representative images of phosphorylated SMAD3 immunohistochemical staining of renal tissues and quantitation using ImageJ. (**F**) Expression levels of phosphorylated SMAD3, SMAD3, and TGF-β1 as determined by western blot analysis and quantitation using ImageJ. Data are presented as the mean ± SEM (*n* = 6); Panels **(A,B)**, *p < 0.05, **p < 0.01, ***p < 0.001 vs. d*b/m*; ^#^ *p* < 0.05, ^##^ *p* < 0.01, ^###^ *p* < 0.001 vs. d*b/*d*b*; Panels **(C–F)**, *p < 0.05, **p < 0.01, ***p < 0.001.

Consistent with the increase of mRNA levels of EMT markers in DN, we found significantly upregulated gene transcription of *Acta2*, *Fn1*, and *Col 4* by ADR treatment while FGF1^ΔHBS^ treatment restored them to normal levels (Figure 5A). Furthermore, there were significant reductions in protein expression of α-SMA, COL 1, and COL 4 associated with FGF1^ΔHBS^ treatment (Figure 5B). Consistent with reduced mRNA expression, FGF1^ΔHBS^ also reduced TGF-β1-positive cells in renal tissues of ADR-treated mice (Figures 5C,D). And the phosphorylation levels of SMAD3 were attenuated by FGF1^ΔHBS^ treatment (Figure 5E). Immune blotting analysis showed that ADR treatment increased TGF-β1 expression and SMAD3 phosphorylation that was significantly restored with FGF1^ΔHBS^ treatment (Figure 5F). These results suggest that FGF1^ΔHBS^ suppresses TGF-β1-mediated renal fibrosis and EMT.

### FGF1^ΔHBS^ Suppressed Podocyte EMT Via Upregulation of PPARγ Under ADR Challenge

**FIGURE 6:**
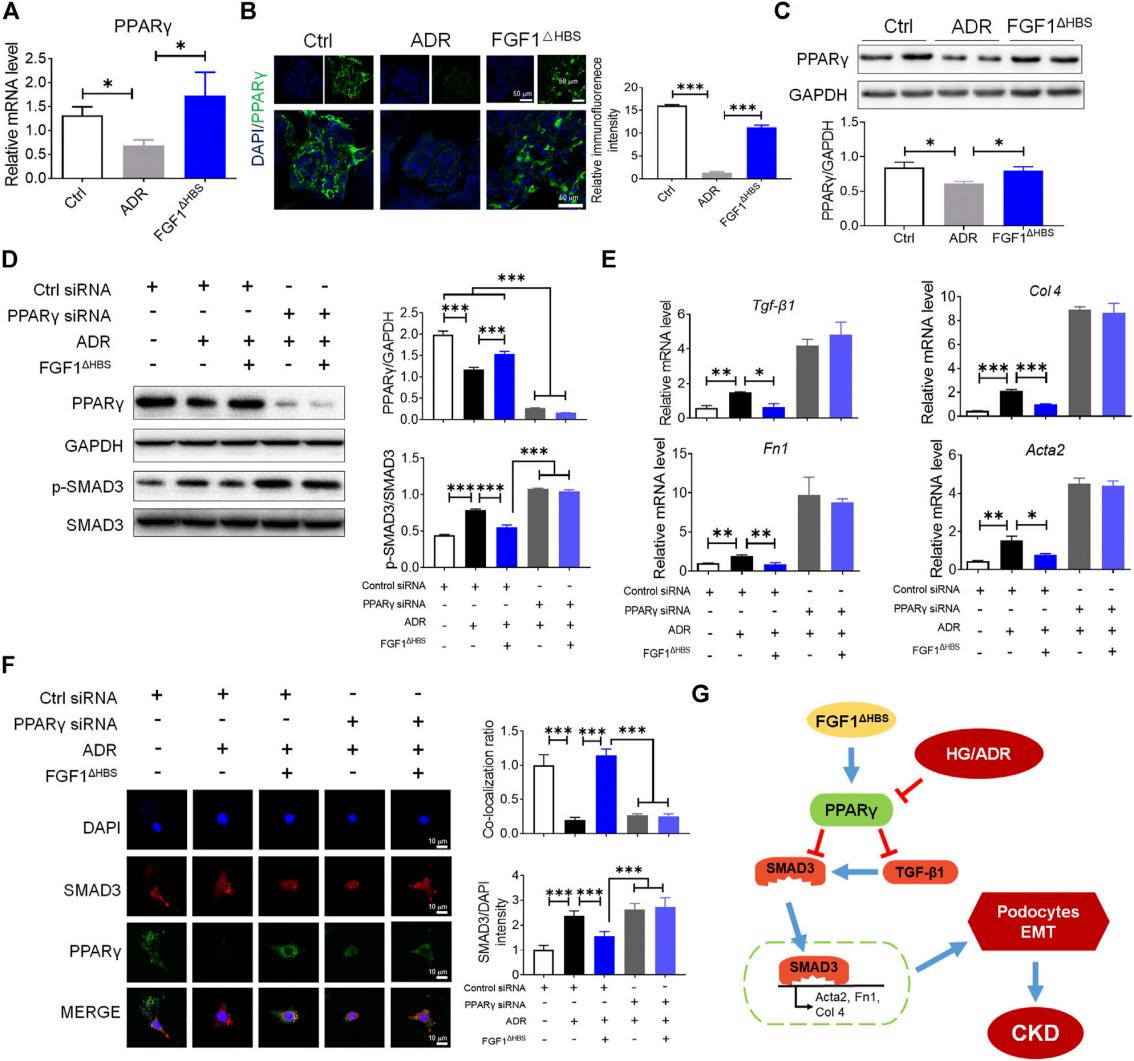
FGF1^ΔHBS^ suppressed podocytes EMT via upregulation of PPARγ under ADR challenge. **(A)** Real-time PCR analysis of PPARγ mRNA expression. **(B)** Representative images and quantitation of immunofluorescence staining for PPARγ. **(C)** Expression levels of PPARγ as determined by western blot analysis and quantitation using ImageJ. **(D)** Phosphorylation levels of SMAD3 and protein expressions of PPARγ as determined by western blot analysis and quantitation using ImageJ. **(E)** Real-time PCR analysis of *TGF-β1*, *Fn1*, *Col 4*, and *Acta2* mRNA expression. **(F)** Representative images and quantitation of immunofluorescence staining of SMAD3 and PPARγ. **(G)** A mechanistic illustration of FGF1^ΔHBS^ protection from diabetes or chemical induced podocyte EMT and CKD. In panels **(A–C)**, Data are presented as the mean ± SEM (*n* = 6). In panels **(D–F)**, data from three independent measurements are presented as the mean ± SEM; *p < 0.05, **p < 0.01, ***p < 0.001.

To determine whether PPARγ mediated the protective effects of FGF1^ΔHBS^ in AN, we investigated the expression of PPARγ using various methods. The mRNA and protein levels of PPARγ were decreased by ADR treatment (Figures 6A–C). FGF1^ΔHBS^ treatment significantly enhanced the transcription and protein levels of PPARγ (Figures 6A–C). Mouse podocytes were used to analyze the *in vitro* protective effects of FGF1^ΔHBS^. As shown in Figure 6D, we found that FGF1^ΔHBS^ upregulated PPARγ expression and suppressed SMAD3 phosphorylation under ADR challenge. The suppression effect was abolished when cells were treated with PPARγ siRNA. ADR increased the expression of pro-EMT genes (*Tgf-β1*, *Fn1*, *Col 4*, and *Acta2*), and FGF1^ΔHBS^ attenuated this induction in a PPARγ-dependent manner (Figure 6E). Consistent with the results of HG treatment, SMAD3 nuclei translocation was decreased, and enhanced interactions between PPARγ and SMAD3 were observed following FGF1^ΔHBS^ treatment (Figure 6F). These results suggest that the protective effects of FGF1^ΔHBS^ against podocyte EMT were independent of glucose control.

## Discussion

The characteristics of CKD where GBM composition is impaired are associated with progressive renal dysfunction, highlighting the importance of podocyte integrity in maintaining normal filtration (Kriz and Lemley, 2015). Activation of TGF-β1/SMAD3 signaling accelerates the overproduction of ECM, promotes podocyte EMT, and participates in the pathogenesis of CKD (Meng et al., 2016). Previously, we reported the protective effects of FGF1 against DN via anti-inflammatory signal transduction (Liang et al., 2018). The present study elucidated a novel mechanism by which FGF1^ΔHBS^ protects podocytes from diabetes- or drug-induced EMT and renal fibrosis. We found that FGF1^ΔHBS^ suppressed TGF-β1 expression and SMAD3 nuclei translocation via activation of PPARγ (Figure 6G).

Inhibition of the TGF-β1/SMAD3 signaling pathway ameliorates non-alcoholic steatohepatitis, tubulointerstitial fibrosis, and myocardium infraction (Chen et al., 2019; He et al., 2020; Okina et al., 2020). Increased EMT of podocytes induced by diabetes and ADR is closely related to end-stage renal disease and glomerular fibrosis. Several lines of evidence demonstrated that TGF-β1/SMAD3 signaling contributes to EMT in podocytes (Kang et al., 2010; Yin et al., 2018). Renal injuries, including mesangial expansion, matrix accumulation, proteinuria, and GBM thickening, were alleviated in SMAD3-null mice treated by streptozotocin (Lin et al., 2009; Yadav et al., 2011). Proteinuria and kidney dysfunction were found in TGF-β1-overexpressing mice (Kopp et al., 1996; Schiffer et al., 2001). In the present study, we found that the upregulation of TGF-β1 induced by diabetic conditions or ADR was inhibited by FGF1^ΔHBS^ treatment. *In vitro* studies showed that FGF1^ΔHBS^ suppressed the expression of EMT markers (*Fn1*, *Atca2*, and *Col 1*). Taken together, these data suggest that the protective effects of FGF1^ΔHBS^ on podocyte EMT are mediated by inhibition of TGF-β1.

PPARγ participates in adipogenesis and exerts diverse effects in other tissues, including liver, skeletal muscle, brain, bone, blood vessels, and kidney (Kawai and Rosen, 2010; Brun et al., 2017). As a transcription factor, PPARγ regulates expression of such genes as *Il-1β*, *Tnf-α*, *Tgf-β*, *Ho-1*, and *Bcl-2* in transactivation- or transrepression-manners (Schmidt et al., 2010; Quelle and Sigmund, 2013; Gross et al., 2017). In addition to transcriptional activity, PPARγ binds to other proteins and regulates their function. Yang et al. (2020) found that decreased interaction between PPARγ and Nur77 resulted in enhanced stability of Nur77 and inhibited metabolic reprogramming in breast cancer. Interactions between PPARγ and NLRP3, β-arrestin-1, and UBR5 regulated inflammatory responses of macrophages, adipogenesis, and endothelial homeostasis (Zhuang et al., 2011; Li et al., 2019; Yang et al., 2021). We found enhanced interactions between PPARγ and SMAD3 and suppressed EMT in podocytes following FGF1^ΔHBS^ treatment. These findings are consistent with a previous report in which a PPARγ agonist reversed pulmonary arterial hypertension (PAH) via inhibition of SMAD3 nuclei translocation in TGF-β1 transgenic mice (Calvier et al., 2017).

FGF1 is a promising agent for the treatment of type 2 diabetes by improving insulin sensitivity. FGF1-null mice displayed an aggressive diabetic phenotype upon a high-fat diet challenge (Jonker et al., 2012; Suh et al., 2014). The glucose-lowering effects of FGF1 or its variants have been reported (Suh et al., 2014; Huang et al., 2017). A high-glucose environment has been suggested to be involved in podocyte EMT. In the current study, we showed that the inhibitory effects of FGF1^ΔHBS^ on podocyte EMT and renal protection were independent of its glucose-lowering activity. Expression of EMT markers was significantly reduced by FGF1^ΔHBS^ treatment *in vivo* and *in vitro*.

## Conclusion

We found inhibitory effects of FGF1^ΔHBS^ on podocyte EMT. The protective mechanism conferred by FGF1^∆HBS^ is mediated by downregulation of TGF-β1 expression and reduced nuclei translocation of SMAD3 via restoration and enhancement of PPARγ expression. We conclude that this is a novel signaling mechanism by which FGF1^∆HBS^ maintains podocyte homeostasis, resulting in protection against decreased glomerular filtration and proteinuria. The findings suggest that FGF1^ΔHBS^ is a promising therapeutic strategy for the prevention of podocyte EMT in CKD.

## Data Availability

The original contributions presented in the study are included in the article/Supplementary Material, further inquiries can be directed to the corresponding authors.
