# Supplementary material for: PPARγ Mediates the Anti-Epithelial-Mesenchymal Transition Effects of FGF1ΔHBS in Chronic Kidney Diseases via Inhibition of TGF-β1/SMAD3 Signaling
Source: Front Pharmacol. 2021 Jun 3;12:690535. doi: 10.3389/fphar.2021.690535 (PMC8209477; doi:10.3389/fphar.2021.690535)
Supplement: Supplementary file 1 [file Table1.DOCX]

**Table S1 Primers used in this study**

| Gene Name | Primer Name | Sequence (5' -> 3') |
| --- | --- | --- |
| Acta2 | Acta2-F | GTCCCAGACATCAGGGAGTAA |
| Acta2 | Acta2-R | TCGGATACTTCAGCGTCAGGA |
| Fn1 | Fn1-F | ATGTGGACCCCTCCTGATAGT |
| Fn1 | Fn1-R | GCCCAGTGATTTCAGCAAAGG |
| Col 4 | Col 4-F | GCTCCTCTTAGGGGCCACT |
| Col 4 | Col 4-R | CCACGTCTCACCATTGGGG |
| Tgf-β1 | Tgf-β1-F | CTCCCGTGGCTTCTAGTGC |
| Tgf-β1 | Tgf-β1-R | GCCTTAGTTTGGACAGGATCTG |
| PPARγ | PPARγ-F | TCGCTGATGCACTGCCTATG |
| PPARγ | PPARγ-R | GAGAGGTCCACAGAGCTGATT |
